# DNA Sequencing Sensors: An Overview

**DOI:** 10.3390/s17030588

**Published:** 2017-03-14

**Authors:** Jose Antonio Garrido-Cardenas, Federico Garcia-Maroto, Jose Antonio Alvarez-Bermejo, Francisco Manzano-Agugliaro

**Affiliations:** 1 Department of Biology and Geology, University of Almeria, 04120 Almeria, Spain; jcardena@ual.es; 2 Department of Chemistry and Physics, University of Almeria, 04120 Almeria, Spain; fgmaroto@ual.es; 3 Department of Informatics, University of Almeria, 04120 Almeria, Spain; jaberme@ual.es; 4 Department of Engineering, University of Almeria, 04120 Almeria, Spain

**Keywords:** DNA sequencing, next generation sequencing (NGS), pyrosequencing, fluorescence, semiconductor, nanopore

## Abstract

The first sequencing of a complete genome was published forty years ago by the double Nobel Prize in Chemistry winner Frederick Sanger. That corresponded to the small sized genome of a bacteriophage, but since then there have been many complex organisms whose DNA have been sequenced. This was possible thanks to continuous advances in the fields of biochemistry and molecular genetics, but also in other areas such as nanotechnology and computing. Nowadays, sequencing sensors based on genetic material have little to do with those used by Sanger. The emergence of mass sequencing sensors, or new generation sequencing (NGS) meant a quantitative leap both in the volume of genetic material that was able to be sequenced in each trial, as well as in the time per run and its cost. One can envisage that incoming technologies, already known as fourth generation sequencing, will continue to cheapen the trials by increasing DNA reading lengths in each run. All of this would be impossible without sensors and detection systems becoming smaller and more precise. This article provides a comprehensive overview on sensors for DNA sequencing developed within the last 40 years.

## 1. Introduction

**Figure 1:**
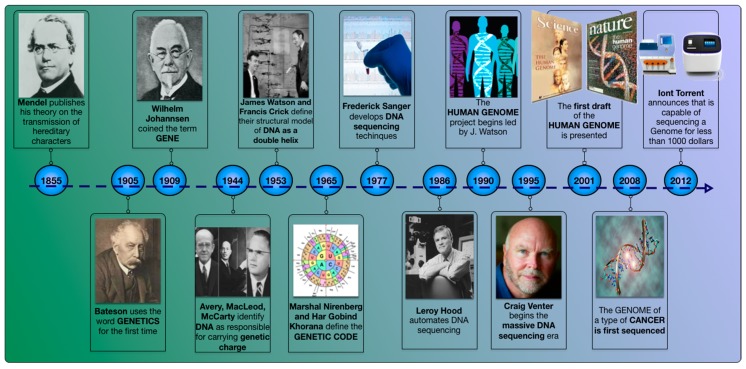
The timeline of DNA sequencing.

**Figure 2:**
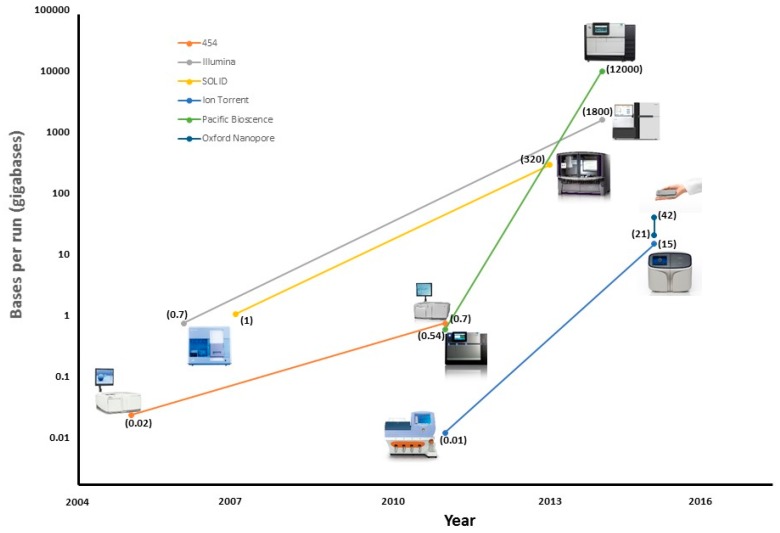
Timeline of the reading capacity of each platform.

Since Avery McLeod and McCarthy’s famous experiment in 1944, in which it was shown that DNA was the transforming principle [1] and, therefore, the material from which genes were composed, the knowledge regarding this molecule has not stopped growing. The next major discovery in the field of molecular genetics was made by Watson and Crick who proposed a double helix as the structural model for DNA [2]. This led them to establish the central dogma of molecular biology. Since then, the interest in determining the primary structure of DNA has been growing, so it can be said that sequencing technology was born as the set of techniques that leads to knowledge about the order in which the four nucleotides—Adenine, Cytosine, Guanine, and Thymine—are present in the DNA. The first organism whose complete genome was sequenced in 1977 was the bacteriophage Phi-X174 [3]. This genome only had 5386 nucleotides distributed in 11 genes, but its sequencing was a great milestone. Since then, the genomes of a large number of species have been sequenced until, finally, the first draft of the human genome was presented in 2001 [4]. Figure 1 shows a timeline of the more important events in the history of DNA sequencing and Figure 2 shows another timeline of the evolution of each platform in the number of bases read per run.

**Figure 3:**
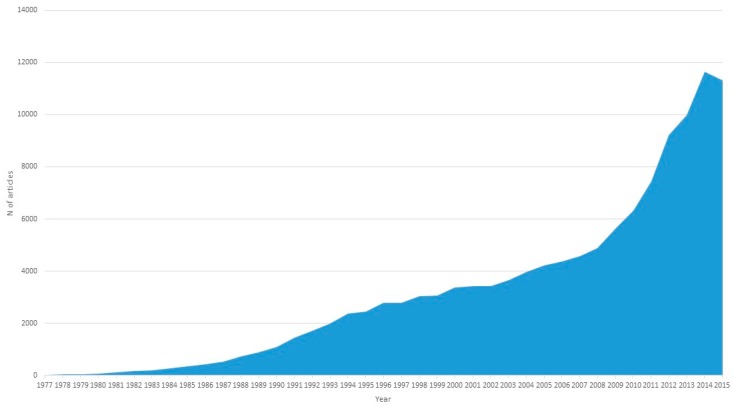
The number of articles published on DNA sequencing annually since 1977.

**Figure 4:**
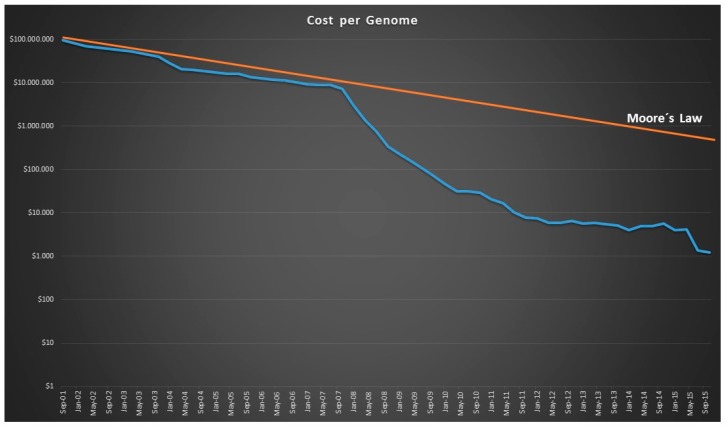
The evolution of the cost of sequencing a complete human genome from 2001 to 2015. The theoretical reduction predicted by Moore’s Law is also represented with a red line.

Since 1977, the number of articles on DNA sequencing has continued to grow, reaching more than 11,000 publications in 2014 and 2015 (Figure 3). In parallel, the cost of sequencing a complete human genome has continued to decline, according to data computed by the National Human Genome Research Institute (https://www.genome.gov/sequencingcostsdata/) (Figure 4). Figure 4 also shows the theoretical reduction imposed by Moore’s Law, which states that the capacity of the hardware used for sequencing doubles every two years. However, what is observed is that the decrease in the cost of sequencing a human genome does not decrease proportionally, as might be expected, but it does so much more abruptly, particularly since 2008. This is due, above all, to the introduction of new generation sequencing (NGS) [5].

**Figure 5:**
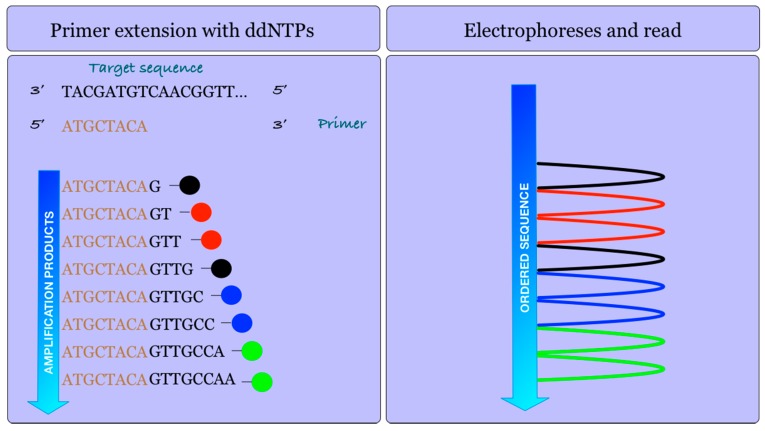
The sanger dideoxy sequencing method or chain termination enzymatic method. Fluorescence-labeled ddNTPs block DNA amplification.

Sequencing of the bacteriophage Phi-X174 and the subsequent DNA sequencing analysis until the completion of the sequencing of the human genome were performed using what is known as the Sanger dideoxy method or enzymatic chain termination method [6]. This method is still widely used today and it is based on the use of dideoxynucleotides (ddNTPs) that block DNA polymerization. The dideoxynucleotides are identical to the deoxynucleotides (dNTPs) that the DNA polymerase enzyme uses to generate a DNA strand from another template strand, with the difference being that the ddNTPs lack a hydroxyl group on the third carbon of the ribose and this causes the enzyme to stop the polymerization of the molecule, since it is not able to find the chemical group to anchor the next nucleotide. In practice, what is done is to feed the reaction catalyzed by the DNA polymerase with a mixture of dNTPs and ddNTPs, so that at each addition of a nucleotide, the enzyme can incorporate either of them randomly (Figure 5). Additionally, ddNTPs are labeled by a fluorophore molecule, so that each time one of them is incorporated, the reaction will stop and the resulting molecule will emit a signal that will report on the last incorporated nucleotide (Adenine, Thymine, Cytosine, or Guanine), since each of them are marked with a different fluorophore. Thus, after an adequate number of cycles of amplification, we will find a number of molecules equal to the number of nucleotides contained in the DNA fragment to be sequenced, differentiating these molecules from each other by a single nucleotide [7]. Next, a capillary electrophoresis is performed with these molecules [8], so that they will be arranged in increasing order of molecular mass, each of which can be identified by the fluorophore attached to the corresponding ddNTPs terminator of the reaction [9]. The detection is performed by a Charge-Coupled Device (CCD) spectral detector [10].

However, due to the approach of ambitious massive sequencing projects, such as the human genome project, it was necessary to develop a new technology that would reduce the costs and the time required to obtain the sequences [11,12]. In this way, New Generation Sequencing (NGS) was born, a high-performance technology based on the parallelization of the sequencing process, resulting in the reading of thousands or even millions of sequences simultaneously [13]. At present, there are eight large massive sequencing platforms (Table 1), which are different from each other in terms of the method of preparing the templates for sequencing, the sequencing reaction itself, and the detection systems used [14]. In addition, each of these platforms can have different equipment, different levels of performance, different numbers of readings and, therefore, a different cost for each sequencing reaction [15].

In any case, there is no doubt that the development of all this technology has necessarily gone hand in hand with new detection systems for signals and sensors whose sensitivity has continued to grow [16]. Increased research on NGS, as reflected in Figure 1, can only be understood from the understanding that the new detection systems have evolved by leaps and bounds. If this had not been the case, the development of DNA reading systems would not have been possible.

## 2. Short-Read Sequencing

### 2.1. 454 Roche Platform

**Figure 6:**
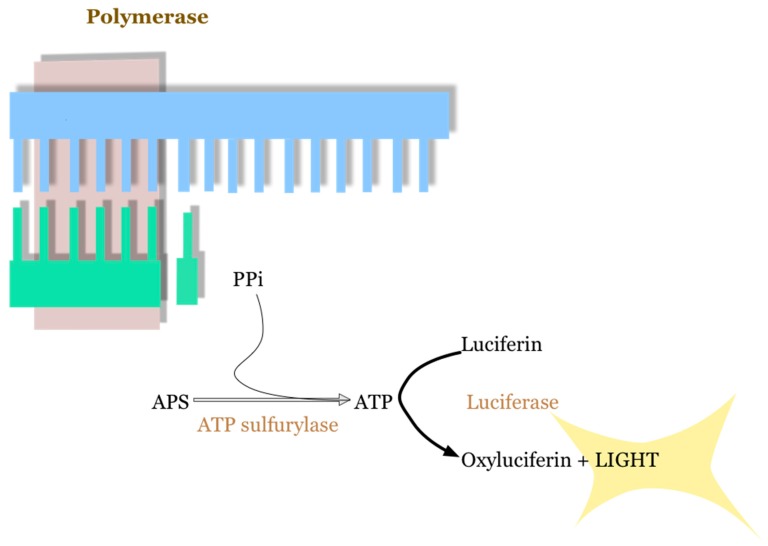
The transformation reaction of luciferin to oxyluciferin, with light release. ATP is generated by ATP sulfurylase from adenosine 5′phosphate (APS), in the presence of pyrophosphate, which is used as a cofactor.

The Roche 454 was the first NGS equipment to be marketed, and its technology is based on pyrosequencing [17,18]. In this case, the sequencing is carried out by a synthetic process so that the reading is performed as nucleotides are incorporated into the template strand replication. Therefore, the methodology is based on the iterative incorporation of each of the four nucleotides. In this platform, the pyrophosphate molecule that is released during the incorporation of the nucleotides in the replication of the template DNA is detected, taking advantage of the fact that the liberated pyrophosphate is proportional to the incorporated nucleotides. The measurement of the pyrophosphates is carried out by the detection of emitted light, which is a byproduct of the transformation of luciferin into oxyluciferin—the reaction is performed by an enzyme called luciferase—and this reaction requires, as a cofactor, the ATP generated by the ATP sulfurylase from adenosine 5′-phosphate (APS), in the presence of pyrophosphate [19] (Figure 6). In addition, the bases that are not incorporated in each cycle are eliminated by the action of an apyrase enzyme, to avoid these residues interfering in later cycles [20]. The reactions are conducted in each of the one million wells that structure the PicoTiterPlate™ (Branford, CT, USA) plates, and the light generated in the reaction—with a maximum wavelength of 560 nanometers—is detected by a CCD camera [21]. That is, the enzymatic cascade that takes place after the incorporation of a dNTP is ultimately responsible for the produced bioluminescent signal.

### 2.2. AB SOLiD Platform

**Figure 7:**
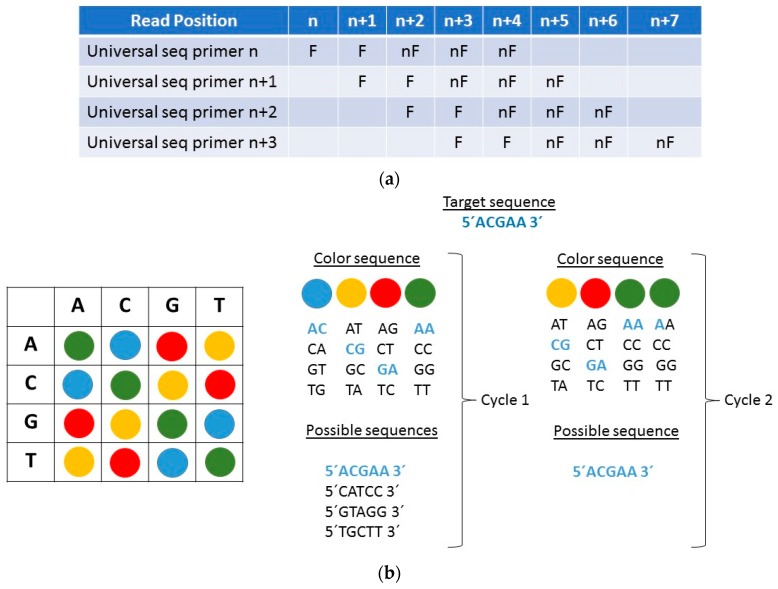
(**a**) Use of the color-space technique in determining the order of nucleotides by sequential ligation of fluorescent probes. F: Fluorescent. nF: non-fluorescent; (**b**) Assignment of colors to nucleotide pairs in sequencing by oligonucleotide ligation and detection.

The SOLiD (sequencing by oligonucleotide ligation and detection) platform, like Sanger sequencing, is based on the detection of fluorescence signals with the difference being that while in Sanger sequencing a fluorophore is used for each nucleotide, in SOLiD sequencing a fluorophore is used for a given combination of two nucleotides. That is, each fluorescence signal represents the binding of two nucleotides. Thus, the raw data obtained cannot be translated into a known nucleotide sequence because each of the four signals refers to a subset of four nucleotide combinations. This methodology is based on the sequential ligation of fluorescent probes [22], so that although only four fluorophores are used for the 16 possible combinations of nucleotides 2 to 2, it is possible to determine which nucleotide occupies each position thanks to the known color-space technique (Figure 7a) [23]. In the SOLiD platform, the ligation and detection of the oligonucleotides is carried out in four steps. In the first step, each fragment to be sequenced hybridizes to one of the 16 labeled probes that have two bases of a known sequence at positions *n* and *n* + 1, followed by a sequence of degenerate bases. In a second step the probe is cleaved, releasing the end to which the fluorophore is bound, and leaving a 5′-phosphate group together with five nucleotides, two of which are of a known sequence. Next, an extension process is carried out, with 10 rounds of hybridization, ligation, and cleavage. Finally, the completion is performed to start the cycle again, but this time in the *n* + 2 position. The fluorescence signal obtained in each measurement should not be proportional to a determined nucleotide, but it will limit the number of possibilities to four, requiring successive cycles of ligation to clear the unknowns [24]. Thus, if in the Sanger sequencing the reading of each position was associated with a fluorescence signal, so that it was automatically translated as soon as it was produced, in the SOLiD sequencing the reading can only be understood in a set of signals [25]. The color space technique was a novelty that introduced the SOLiD platform and this is only used by it. In this technique, in contrast to the base space technique of Sanger sequencing, each signal does not represent one base but two bases in a row. Each nucleotide pair receives a certain color, but as can be seen in Figure 7b, the color matching for each nucleotide pair is not random. Reverse (e.g., AG and GA), complementary (e.g., AG and TC), and complementary reverse (e.g., AG and CT) couples are shared by the same fluorophore. By having an ACGAA sequence (Figure 7b), the first probe will have AC in its first two positions, the second probe will have CG, the third probe will have GA, and the fourth probe will have AA. However, there are up to four possible combinations, so we need a second reading. Considering this, the only possible sequence is the one that throws an ACGAA reading. The ligation, detection, and cleavage reactions are performed as many times as nucleotides have the sequence to be determined.

A variation of this method is used by the Complete Genomics (CG) platform, created in 2006 and acquired in 2013 by the Chinese company BGI-Shenzhen. This is presented as an ideal platform for the detection of variants in large-scale genetic studies, such as in projects related to the human genome, given its high precision and low cost [26]. The two main novelties presented with respect to the other sequencing platforms are the use of DNA nanoboles and the ligation technique by the combinatorial probes anchor ligation, cPAL. DNA nanoboles (DNBs) are fragments of circularized DNA template, following the fragmentation with restriction enzymes by the use of directional adapters, so that each one of them has different density, size, and even affinity properties. Each DNBs contains many copies of the original DNA template.

**Figure 8:**
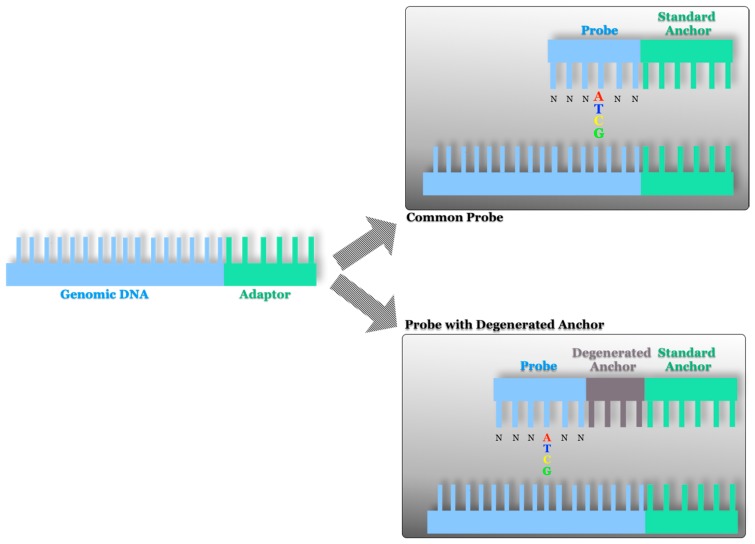
Combinatorial probe anchor ligation in the CG platform. The probes contain degenerate and fluorophore-tagged DNA fragments attached to the standard anchor sites.

cPAL consists of the use of nonamer probes containing degenerate and fluorophore-tagged DNA fragments attached to the standard anchor sites to read the bases adjacent to the degeneracy by ligation of these probes (Figure 8). This is achieved by moving from readings of 6–7 base pairs to readings of 11–12 base pairs. In this way, the CG platform is the only one in which the reading of the DNA sequences is carried out in solution. The ligation performed in this methodology is known as unchained, since with the detection of each probe, the system starts a new cycle from zero, minimizing the background [27] because the unligated probes are washed away. In this platform the flow cell is imaged by simultaneous high speed detection of the four colors.

The sequencing by ligation used by the SOLiD and CG platforms presents the enormous advantage of offering a very high precision in the reading of the sequences (Table 2). This is because each position is read several times and with different probes. In contrast, they do present some important drawbacks such as the short reading length obtained or the long time necessary to obtain the results.

### 2.3. Illumina Platform

**Figure 9:**
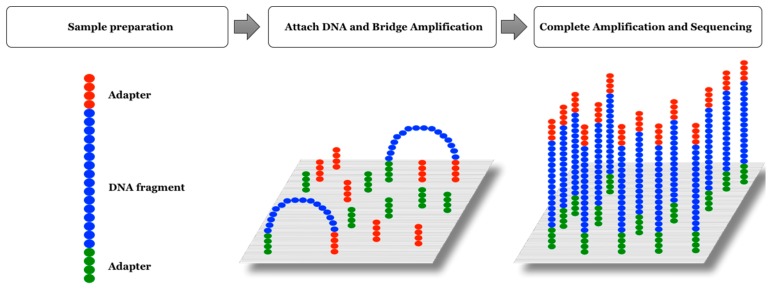
Illumina sequencing. The fragments are attached to primers immobilized on a solid surface, performing a bridge-amplification and generating clusters of DNA with identical molecules.

In this platform, DNA sequencing is carried out by fluorescence-labeled nucleotide analogs acting as reversible terminators of the amplification reaction [28]. The idea is similar to that developed to carry out the Sanger sequencing, with the difference being that in the Illumina platform the blockade of DNA polymerization is reversible and in the sequencing of Sanger this is irreversible. Another different feature of this technology is that the clonal amplification in vitro to multiply the number of molecules to be sequenced is conducted by means of bridge PCR. In this platform, the fragments are joined to primers immobilized on a solid surface, performing an amplification in situ, generating clusters of DNA with identical molecules [29] (Figure 9). In each cycle, the four nucleotides of reversible termination are simultaneously added and incorporated by the polymerase they complement. These nucleotides are chemically blocked—by substituting the 3′-OH group for a 3′-*o*-azidomethyl group—to prevent the polymerase from incorporating more than one nucleotide in each cycle. Upon incorporation of a nucleotide, a fluorescence signal is output which is measured by total internal reflection fluorescence (TIRF) using various laser channels. Concerning the next cycle, the nucleotides that have not been incorporated are washed and the chemical blockade of the 3′ end is removed through the use of tris-(2-carboxyethyl)-phosphine, to continue the synthesis of the chain [30]. For this reason, they are cyclic reversible termination nucleotides. Once the fluorescence signal is collected, a new cycle begins, repeating this dynamic until the sequencing of each fragment is finished. In summary, we could say that the sequencing reaction is carried out in three steps: addition of nucleotides, imaging, and regeneration of 3′-OH by fluorophore cleavage.

As indicated above, the detection system used in the Illumina platform is the Total Internal Reflection Fluorescence (TIRF), also known as evanescent wave microscopy. Its main advantage over other systems is that it is able to detect the fluorescence of molecules that are very close to a solid surface (glass or plastic), and is highly selective [31]. By means of TIRF, it is possible to illuminate a very thin layer of less than 100 nm in depth, avoiding the excitation of other fluorophores that may be near but whose emission is not required for the measurement [32].

This same detection system is used by the Qiagen GeneReader platform, which was launched on the market in 2015, after Qiagen acquired the Intelligent BioSystems CRT platform. The great contribution of this new platform, and its great advantage, is that it is presented as an all-in-one platform [33], from the preparation of the samples to the analysis of the results [34]. Another difference, methodological in nature, is that the group that is blocking the 3′-OH of the nucleotides is not an O-azidomethyl, as was the case in the Illumina platform, but an O-allyl, and that the regeneration of the 3′-OH is performed with a mixture of palladium and P(PhSO_3_Na)_3_ (TPPTS) in the GeneReader platform, while in the Illumina platform the regeneration was carried out with the reducing agent tris (2-carboxyethyl) phosphine (TCEP). Moreover, both the sequencing methodology by cyclic reversible termination and the detection of the signal by total internal reflection fluorescence (TIRF) are identical to that of the Illumina platform.

At present, Illumina’s sequencers are the most widely used in the development of massive sequencing projects. This is not only due to their high precision in sequencing and the low cost of the Gb (Gigabytes) obtained (Table 3), but also because they have a great variety of equipment in the market that can adapt to the needs of each project. This ranges from small medium-performance bench-top units, such as the MiniSeq, to mega equipment used for sequencing projects of whole genomes in populations, such as the HiSeqX.

### 2.4. Ion Torrent Platform

**Figure 10:**
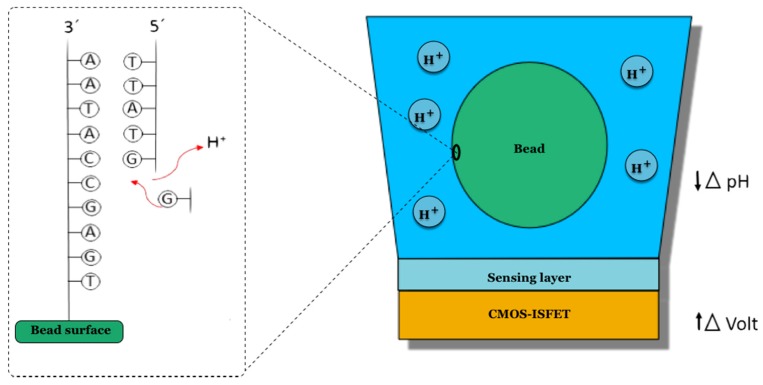
In the Ion Torrent platform, the chip is the sequencer. Each well of the chip acts as a pH meter that is able to detect changes in the concentration of H+ produced in DNA polymerization.

The Ion Torrent platform is based on semiconductor technology and it was the first to use non-optical sensors [35]; so for the first time, the technology used for DNA sequencing eliminates both optical scanning and dNTPs attached to fluorophores [36]. The process that is performed is the same as the one used in the construction of integrated circuits in computer chips. This is based on the complementary metal-oxide-semiconductor (CMOS) process [37], to monitor the detection of protons (H^+^) in DNA synthesis, when the incorporated dNTP is complementary to the nucleotide of the template chain being copied [38]. The great success of this platform consists of the integration of a chip that has millions of CMOS sensors in its matrix, so that the compilation of all the data can be performed in an inexpensive and simple way [39]. The second major innovation of this platform was the introduction of an electro-chemical ISFET (ion field sensitive transistor) sensor at the bottom of each well [40], which act as a pH meter that is sensitive to changes in H^+^ concentration (Figure 10).

To perform sequencing on the Ion Torrent platform, the DNA template is presented on the surface of a sphere (or bead) obtained by a PCR emulsion [41]. Subsequently, the addition of a single nucleotide will occur one at a time so it is not necessary to block the dNTPs, as in the case of cyclic reversible CRT sequencing. In other words, unlike other platforms, in this case the nucleotides that are used are not chemically modified. Thus, when the nucleotide that has been added is incorporated by the polymerase into the DNA strand being synthesized, a proton is released and this is detected by the CMOS-ISFET sensor, generating a signal that is sent to a computer that will process it.

Unlike the Illumina platform, the Ion Torrent platform does not have a large number of devices on the market. Even so, their sequencers present some versatility since there are several types of chips (which is the support in which the sequencing reaction is carried out) that adapt to the dimensions of each project. Thus, chips with yields ranging from 50 Mb to 15 Gb can be found. For chips with the highest throughput, the run time is not more than 7 h, and for lower throughput, the run time is about 2 h. Therefore, this short time is one of the main advantages of this platform (Table 3). Thanks to these characteristics, the Ion Torrent platform is finding its niche market in the analysis of gene groups (diagnosis of polygenic diseases, metagenomics, etc.), i.e., in clinical sequencing.

## 3. Single-Molecule Real-Time Long Read Sequencing

### 3.1. Pacific Bioscence Platform

Previous platforms performed sequencing from small DNA fragments—up to 1000 bp on the 454 platform—that were processed and modified according to the method of reading, whether by a ligation reaction or by a synthesis reaction. However, there are new platforms, known as third generation platforms [42], whose objective is the sequencing of simple molecules in real time. These platforms take advantage of advances in the field of nanotechnology, although the way in which they perform the sequencing reaction is different. Their main advantage is that library preparation is not necessary and sequencing reagents are not needed [43,44].

**Figure 11:**
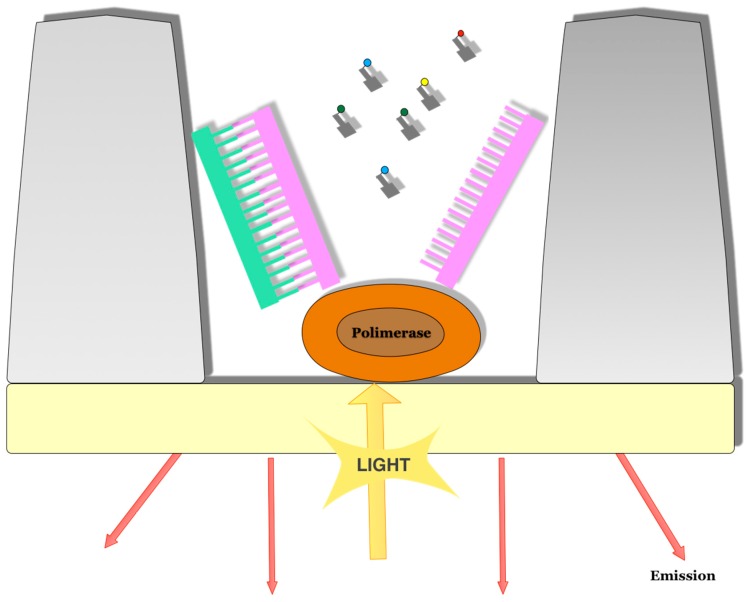
Polymerase fixed to the bottom of an individual well. In the Pacific Bioscience platform, the DNA moves generate signals because of the incorporation of phosphate-labeled nucleotides.

The Pacific Bioscence platform was the first to carry out this third-generation sequencing, and DNA is sequenced by a single-molecule real-time (SMRT) synthesis of a single molecule [45]; this is, to this day, the platform most often used to carry out this type of sequencing. Whereas in the systems seen so far for the synthesis (SBS) of small fragments, the DNA was fixed so that the polymerase could be moved along it to perform the synthesis of the new chain; in the case of the Pacific Bioscence platform, the polymerase is the one that is fixed to the bottom of an individual picolitre well with a transparent bottom, so that the DNA has mobility (Figure 11). In this platform, the DNA sequencing is carried out thanks to nanosensor technology called ZMW, zero-mode waveguide [46], that detects the signal generated by the incorporation of phosphate-labeled nucleotides to the well, where a single DNA polymerase replicates the DNA. The sequencing takes place in the ZMW SMRT cell. ZMW are devices whose size prevents the propagation of light. As a result, visible laser light does not pass through the ZMW sensors, so that marked nucleotides that are not incorporated by the polymerase and are located above these sensors do not contribute to the measured signals. These only fluoresce when they are incorporated by the enzyme and diffuse through the sensor’s ZMW. The incorporation of each dNTP is continuously displayed with a laser and a camera system that records the signal emitted during the incorporation into the lower part of the ZMW. Imaging is possible thanks to the action of a powerful optical system that illuminates individual ZMWs with red and green laser beams from the bottom of the SMRT cell and the existence of a parallel confocal system that detects the fluorescence signal from the incorporated nucleotides [47].

### 3.2. Oxford Nanopore Platform

**Figure 12:**
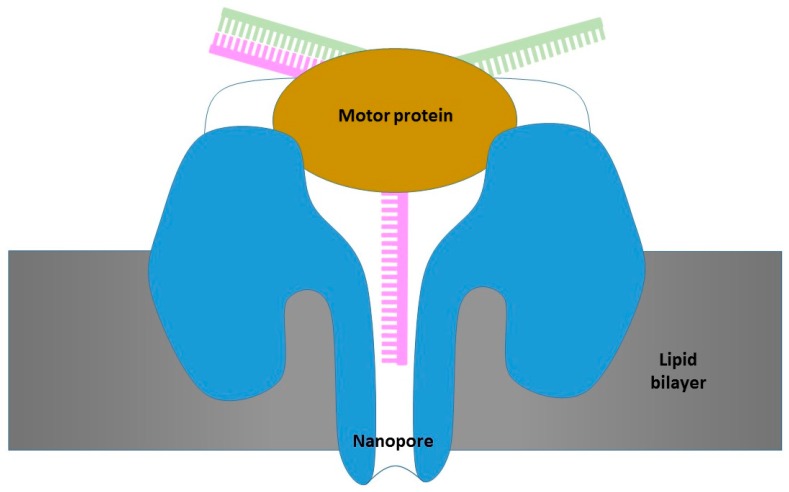
Channels used by the Oxford Nanopore platform to sequence DNA. The passage of DNA through the nanopore produces alterations that are measured thanks to the detected voltage changes.

Another platform being used for real-time DNA sequencing from a single molecule is the Oxford Nanopore platform, which uses nanosensors that form channel structures and that carry the sample to a sensor that allows for the detection of each nucleotide residue present in the DNA strand [48,49]. This technology is based on tunneling by creating pores to separate two compartments [50]. Similar structures are being used for the detection of specific DNA sequences [51]. In this case, it is the molecule that traverses the pore that causes a temporary change in the potential between the two compartments, and this change allows its identification [52] (Figure 12). Thus, instead of using a secondary signal such as light, color, or pH to detect read DNA, the nanopore platform directly detects the composition of a DNA template [53]. The DNA molecule crosses the pore thanks to the action of a secondary motor protein, producing an alteration in the potential between both sides of the pore. These shifts in voltage are characteristic of each DNA sequence [54,55]. The variation observed in the measured voltage is not only a consequence of the potential change produced by the passage of a DNA fragment, but also of its duration, so that this measure can be interpreted as a particular k-mer sequence. A flow cell structure is composed of an application-specific integrated circuit (ASIC) chip and each one has 512 individual channels that are capable of sequencing more than 60 bp per second. Considering the fact that this technology uses unmodified DNA, it has the advantage of yielding results very quickly from minimal starting quantities. At present, this platform works with both nanopores obtained from genetically engineered proteins and fully synthetic nanopores [56].

The first prototype of this platform was the Minion and it was launched on the market in 2014. This one attracted a lot of attention because of its small size and simplicity. Another advantage is that it does not determine the DNA sequence through secondary elements such as light or H^+^ concentration, but it does so directly and in real time.

However, despite the promising potential caused by the launch of simple molecule sequencing platforms in real time, these still have the great disadvantage of low accuracy. In the future it will be necessary to combine the best aspects of each platform to have a sequencer that offers complete genome sequences quickly, cheaply, and simply. At that time, terms like personalized diagnosis, genomic medicine, or completely individualized medical treatments will become a reality.

## 4. Conclusions

This manuscript aims to highlight the importance of DNA sequencing sensors describing the state of art of the topic and presenting the availability of its methods and platforms. Multiple short-read sequencing platforms were analyzed: 454 Roche, AB SOLiD, Complete Genomics, Illumina, GeneReader, and Ion Torrent. Additionally, two single-molecule real-time long read sequencing platforms were analyzed: Pacific Bioscence and Oxford Nanopore. One can envisage that incoming technologies, already known as fourth generation sequencing, will continue to cheapen the cost of trials while increasing DNA reading lengths in each run. All of this would be impossible without sensors and detection systems that are becoming smaller and more precise. The evolution of DNA sequencing sensors along the last 40 years reveals some very impressing results and opens up new perspectives for science.

Knowing the sequence of a DNA fragment has multiple uses, such as performing phylogenetic studies, diagnosing diseases [57], or controlling pathogens. The tools with which all these applications can be developed are biosensors [58] or DNA sensors. Thanks to these, a particular sequence of genetic material or an enzymatic activity can be detected in a complex sample. To date, a multitude of biosensors have been successfully used for applications such as food, for the control of pathogens, allergens, or toxins [59]. However, the world of biosensors still faces many challenges. The first one is that the test sample usually contains a very low DNA concentration, therefore a previous amplification is necessary by means of PCR or another strategy. Other problems are nonspecific amplifications, which give rise to false positives, or the technical difficulties inherent in the use of nanomaterials. In this sense, the use of graphene is being presented as a good solution [60] due to its physicochemical properties, such as its excellent conductivity or high mechanical resistance. Another difficulty that occurs in the use of biosensors is in the molecular recognition by natural receptors. In this case, the use of Molecularly Imprinted Polymers (IPM) helps to reduce this problem, providing convenient solutions [61]. In any case, the use of biosensors is spreading and is expected to maintain its exponential growth to reach a world with ubiquitous sensors. Therefore, the combination of improvements in both the molecular field and the study of new materials seem fundamental.

## Figures and Tables

**Table 1:** Massive sequencing platforms presently available.

Methods of DNA Sequencing			Platform
Short-read sequencing	Sequencing by ligation		AB SOLiD (Thermo Fisher)
			Complete Genomics (BGI)
	Sequencing by synthesis	Cyclic reversible termination (CRT)	Illumina
			GeneReader (Qiagen)
		Single-nucleotide addition (SNA)	454 Roche
			Ion Torrent
Single-molecule real-time long read sequencing			Pacific Bioscence
			Oxford Nanopore

**Table 2:** Comparison of the different sequencing platforms. The data shown refer to the most favorable conditions for each platform.

Platform	Read Length (bp)	Accuracy (%)	Run Time	Bases Per Run (Gb)	Cost/Gb
454 Roche	1000	99	24 h	0.54	$10,000
SOLiD	75	99.9	7 d	520	$10
Illumina	300	99.9	3 d	1800	$10
Ion Torrent	400	99	2 h	15	$100
Pacific Bioscence	20,000	90	3 h	12,000	$600
Oxford Nanopore	10,000	90	2 d	42	$1000

bp, base pairs; Gb, gigabase pairs; h, hours; d, days.

**Table 3:** Pros and cons of each platform.

Platform	Pros	Cons
454 Roche	Long reading length. Low analysis time. Low cost for small studies	High error rate in homopolymers. Low performance. High instrumental cost. High cost per Gb data
SOLiD	High throughput. Low cost per Gb data. High accuracy	Short reading length. High instrumental cost
Illumina	High throughput. Low cost per Gb data. High accuracy	Short reading length. High instrumental cost
Ion Torrent	Low instrumental and operational cost. Short execution time. Very simple machine	Error rate not very good. Intermediate cost per Gb data. More hands-on time
Pacific Bioscence	Longest reading length available. Short instrument execution time	High error rate. High cost per Gb data. Many methods are still under development
Oxford Nanopore	Small, portable, and low cost instrument	High error rate. Biased errors. High cost per Reading

Gb, gigabase pairs.
